# Cost analysis of chronic obstructive pulmonary disease (COPD): a systematic review

**DOI:** 10.1186/s13561-021-00329-9

**Published:** 2021-08-17

**Authors:** Celia Gutiérrez Villegas, María Paz-Zulueta, Manuel Herrero-Montes, Paula Parás-Bravo, María Madrazo Pérez

**Affiliations:** 1 IDIVAL, Research Institute Marqués de Valdecilla. C/ Cardenal Herrera Oria s/n, 39011 Santander, Spain; 2 Faculty of Nursing, University of Cantabria, Avda. Valdecilla s/n. C.P, 39008 Santander, Cantabria Spain; 3 IDIVAL, GI Derecho Sanitario y Bioética, GRIDES. C/ Cardenal Herrera Oria s/n. C.P, 39011 Santander, Cantabria Spain; 4 IDIVAL, Grupo de Investigación en Enfermería. C/ Cardenal Herrera Oria s/n. C.P, 39011 Santander, Cantabria Spain

**Keywords:** Systematic review, Pulmonary disease, Chronic obstructive, Cost of illness, Health care costs, Investments

## Abstract

**Background:**

Chronic Obstructive Pulmonary Disease (COPD) is a treatable disease with a high prevalence, and high morbidity associated with significant socioeconomic costs.

**Objective:**

To carry out a systematic review of the literature to analyze the main cost studies associated with COPD, in order to determine the main factors that influence the costs of the disease.

**Methods:**

Searches were conducted in PubMed, SCOPUS and Web of Science databases for cost studies on COPD published in English, between the years 2015 and 2020. The search terms were “COPD” OR “pulmonary disease, chronic obstructive”, “cost*” OR “cost of illness”, “economic impact” AND “burden of disease”. The inclusion criteria included the identification of reported cost of the disease, economic burden, medical care expenses or use resources for COPD, the methodology used, data sources, and variables studied.

**Results:**

18 publications were analyzed: 17 included direct health costs, 6 included direct non-medical costs, 12 analyzed indirect costs and two reported intangible costs. Most of the studies reported data for developed and European countries, with direct costs being the most studied. Trends were observed in multiple studies of direct and healthcare costs for European countries measured by patient and year, where the higher costs were associated with more severe COPD and a frequent history of exacerbations. The highest costs reported corresponded to hospitalizations and the associated pharmacological treatment. The importance of the loss of productivity and premature retirement within the profile of the COPD patient was also highlighted as the main generator of indirect costs of the disease.

**Conclusion:**

COPD generates substantial costs for the health system, mainly related to moderate to severe stages and the exacerbations and complications entailed. It is important to strengthen health systems with monitoring, evaluation and health education models that allow these patients to remain stable to avoid decompensation and subsequent hospitalizations.

## Introduction

According to the Global Initiative for Chronic Obstructive Lung Disease (GOLD), COPD is defined as *“a common, preventable, and treatable disease that is characterized by persistent respiratory symptoms and airflow limitation that is due to airway and/or alveolar abnormalities usually caused by significant exposure to noxious particles or gases”* [1], tobacco being the main cause detected [2].

The clinical manifestations of this heterogeneous disease are generally identified in people between the age of 40–50 years, although symptoms may appear earlier. The main symptoms associated with the disease are dyspnea, chronic cough, and expectoration. These symptoms can often mislead the diagnosis, as they can be confused with other chronic respiratory pathologies with a similar course, such as asthma. The literature generally identifies four phenotypes or clinical forms within the disease that determine different treatments: non-acute, with emphysema or chronic bronchitis, COPD-Asthma, acute with emphysema and acute with chronic bronchitis [2].

The natural history of COPD includes episodes of exacerbation, which become common in the moderate and severe stages of the disease. These episodes increase morbidity-mortality, health system resource utilization (increased costs) and lead to loss of productivity and deterioration of the health-related quality of life (HRQoL) of the patients who suffer from them [2, 3]. Exacerbations are defined as an acute episode of clinical instability characterized by a worsening of respiratory symptoms” [2]. Patients with COPD experience an average of one to four exacerbations per year; however, the intensity and duration varies from patient to patient, making it difficult to quantitatively estimate their exact consequences [2]. They are mainly related to respiratory infections or environmental stressors, but sometimes they can also be linked to the presence of other concomitant pathologies, which can produce a greater aggravation of symptoms and, consequently, greater difficulty for the patient’s recovery [4].

Epidemiologically, COPD is identified as the third leading cause of death worldwide, besides representing one of the main causes of increased morbidity, inducing an economic and social burden that is currently on the rise. The morbidity and mortality identified vary from one country to another and from one population group to another, which is why the existing prevalence data widely differ [1].

### Study justification

The main usefulness of cost-of-illness studies is based on the identification and measurement of part of the financial impact of the disease, at the health, social and work levels, helping health system managers with limited budgets and resources to cover a set of unlimited needs and to make a cost-effective allocation between the investment options that exist and are presented to them in their day-to-day activity.

In the case of highly prevalent chronic diseases, it is essential to quantify the social and financial magnitude of the disease in all spheres (direct and indirect costs, health and non-medical costs, labor losses and intangible costs). This will improve the ability to design, prioritize, and apply preventive and health promotion measures, investments in public health or social policies and protocols and care guidelines in the most equitable and efficient way possible. Furthermore, this will also allow constant updating to prevent current practices from becoming obsolete, inefficient, and costly because they are not adapted to the reality of the disease.

Various international studies have been carried out to try to estimate the cost of COPD to society or to the health care system, in different manners and applied to different processes of the disease and with highly variable study designs, thus obtaining heterogeneous results. This variability in the methodology used by the different authors makes it difficult to extrapolate results and conclusions, which can lead to a failure to make the most appropriate and cost-effective decisions for managing a disease with an ever-increasing prevalence in society. In view of the wide variety of estimates, it is important to develop a guide that provides a homogeneous estimate and includes comparable data.

The principal aim of this systematic review was to identify the main factors that lead to increased costs in COPD patients.

## Methods

A systematic review of the literature on COPD-associated cost studies was conducted and served as the basis for the design of the overall structure of this systematic review. The studies were identified and selected by conducting a literature search using three electronic databases: PubMed, SCOPUS, and Web of Science.

The terms included in the thesaurus of the National Library of Medicine of the United States (Medical Subject Headings, MeSH) were used: “COPD or “pulmonary disease, chronic obstructive”, “cost*” or “cost of illness”, “economic impact” and “burden of disease” in the title or abstract fields to retrieve potentially relevant publications in the last five years (January 1, 2015 to July 30, 2020). The search strategy was limited to publications made in English and Spanish. The results were complemented with a new review with free-text language with keywords of the present monograph and filtering in the last five years in the Google Scholar database after which 36 articles were selected (Table 1).

**Table 1:** Literature search strategy: PubMed, Scopus, Web of Science

Database	Search terms	Language	Results
PubMed	“COPD” AND “cost*” AND “economic impact”	English	35
PubMed	“COPD” AND “cost of illness” AND “burden of disease”	English	29
PubMed	“COPD” AND “burden of disease” AND “cost*”	English	12
Scopus	“COPD” AND “cost*” AND “economic impact”	English	20
Scopus	“COPD” AND “burden of disease” AND “cost*”	English	19
Scopus	“COPD” AND “cost of illness”	English	134
Web of Science	“COPD” AND “cost*” AND “economic impact”	English	44
Web of Science	“COPD” AND “burden of disease” AND “cost*”	English	13
Web of Science	“COPD” AND “cost of illness”	English	17

**Source.***Own elaboration*

The inclusion criteria consisted of systematic review or original research, meeting the following conditions:

1. Reported cost of illness, economic burden, health care expenditures, or use of resources for COPD.
2. Development of the methodology used for its elaboration.
3. Identification of information on data sources and study variables.
4. The study population included patients with the disease under study: COPD.
5. Studies published in the last five years (January 1, 2015 - July 30, 2020).
6. Publications in English and Spanish.

**Fig. 1:**
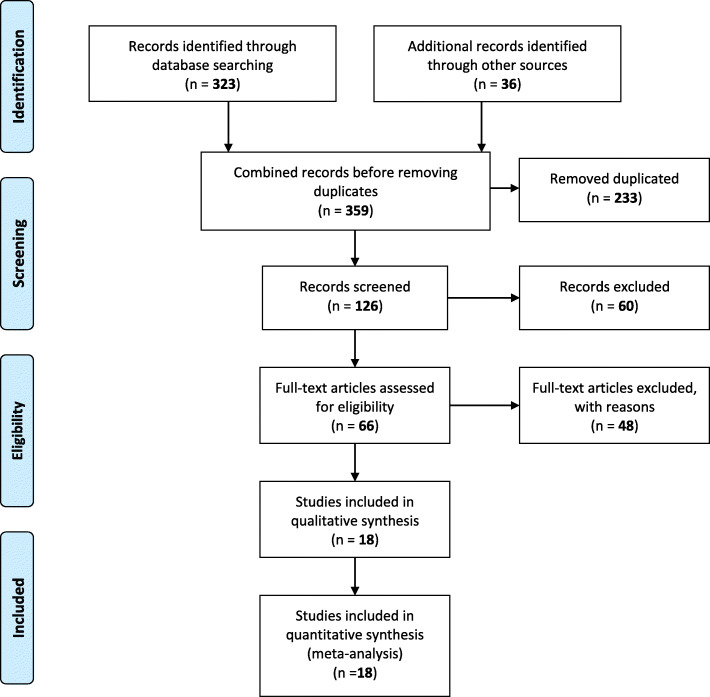
Flow diagram of literature search

Prior to conducting the literature search, a review protocol was developed that included a timeline, the research question, inclusion criteria for the articles to be selected and a table for extracting data from the studies. Figure 1 shows the procedure carried out, following the guidelines proposed in the PRISMA Guide [5]. Thus, the selection process was divided into four parts: identification, screening, selection and inclusion. Identification was carried out by means of the research question in the different databases mentioned above. This phase yielded 359 results. After eliminating duplicate articles (n:233) and exploring the literature, we proceeded to the screening phase, in which the 126 titles of all articles (359–233) were examined and, following the aforementioned inclusion criteria, those that were considered not relevant were discarded (n:60). Those which were doubtful proceeded to the third phase (n:66). One of the limitations found and analyzed later lies in the high number of duplicated articles, probably due to a failure related to the level of hierarchy when establishing the combination between search terms. But nevertheless, to facilitate the management of the volume of articles, the selection phase was divided into two sub-phases; a preliminary phase in which the title and abstract of each of the studies were reviewed for mention of the target population of the study and the inclusion criteria were applied. In this manner, the 66 articles mentioned above were analyzed, after which 48 were discarded, subsequently, in a second phase the studies were assessed as eligible based on the full text. Finally, the remaining 18 articles were included in the review and are presented in this manuscript.

## Results

This review highlights the significant economic burden of COPD. Methodological heterogeneity was found in the studies included in the review. The different authors considered different variables to estimate direct healthcare costs, direct non-medical care costs, indirect costs and intangible costs, and even used different quantification methodologies and different economic conditions, currencies, and billing years. In addition, the variability among the different health systems in which the various studies included in this review are framed must also be considered. In summary, the studies used different approaches to estimate resource consumption. Although each method is valid and appropriate and there is no standard formula to be applied homogeneously in each study, the resources allocated to the disease were identified differently, which can generate disparities in the cost estimation.

This disparity leads to a wide variability of results for the cost of COPD disease. Each perspective provides useful and valid information on costs for a particular group (different samples with different patient sociodemographic and disease-related characteristics), although this makes comparison of results difficult.

First, the most relevant characteristics identified in the eighteen selected articles are presented. The studies selected in this systematic review included the following designs: cohort study (*n* = 11), systematic review (*n* = 5) and cross-sectional study (*n* = 2). These studies were published between 2016 and 2020, thus presenting the most updated data on the subject under study. The monograph is based on a global setting; therefore the analyzed articles were conducted mostly in Europe, but also Asia and America. One of the most important aspects when carrying out a cost study and analyzing its results is to identify the perspective presented by each study. Thus, the present systematic review is made up of studies with a societal perspective (9 articles), followed by the perspective of the National Health System (4 articles), that of the patient (1 article) and that of the public decision-maker (1 article). Finally, regarding the main objective of the research studies analyzed, most studies aim to evaluate the economic and social impact and burden of COPD in the different health systems and societies where the studies are conducted, as well as to identify the main factors that increase the cost of the disease. These are the same objectives sought by the present monograph. See Table 2.

**Table 2:** Selected articles and their relevant characteristics: year of publication, study design, country, year of data, perspective, and principal study aim

Article	Year of publication	Design	Country	Year of data	Perspective	Principal aim
**Foo J et al.** [6]	2016	Cross-sectional study	England	2012–2013	Society	To assess the economic impact of COPD based on an international survey of COPD patients.
**Wacker M. E et al.** [7]	2016	Cohort study	Germany	2010–2013	Society	To investigate COPD medical overuse, work absenteeism and resulting costs within the German COSYCONET cohort.
**Chen W et al.** [8]	2017	Cohort study	Canada	1996–2012	NHS*	To examine the impact of comorbidities on excess direct medical costs in patients with COPD.
**Tachkov K et al.** [9]	2017	Cohort study	Bulgaria	2014–2015	Patient	To calculate the economic and social burden of COPD in Bulgaria generated by lost productivity due to absence from work, early retirement, reduced productivity and DALYs.
**Wacker M. E et al.** [10]	2017	Cohort study	Germany	2010–2013	Society	To analyze the association of frequent comorbidities and common symptoms with the direct and indirect annual costs of COPD patients.
**Quang Vo T et al.** [11]	2018	Cohort study	Vietnam	2015–2017	NHS	To explore the direct medical costs associated with COPD and identify the key factors of disease management costs.
**Lakiang T et al.** [12]	2018	Cross-sectional study	India	2014–2015	Society	To study the economic impact (direct and indirect costs) of COPD patients receiving treatment in a tertiary hospital in South India.
**Lisspers K et al.** [13]	2018	Cohort study	Sweden	2000–2014	Society	To assess the direct and indirect costs of COPD in Sweden in relation to time, age and stage of disease.
**Merino M et al.** [14]	2018	Cohort study	Spain	2015	Society	To estimate the socioeconomic impact of COPD in Extremadura (Spain) in 2015.
**Patel JG et al.** [15]	2018	Cohort study	USA	2007–2010	Society	To measure the impact of COPD by calculating direct and indirect incremental costs.
**Stafyla E et al.** [16]	2018	Cohort study	Greece	2014–2015	Public decision maker	To estimate the annual cost of the COPD patient during the maintenance phase and to explore relationships between cost and disease severity.
**Zhu B et al.** [17]	2018	Systematic review	China	Until 2015	Society	To quantify the burden of COPD in China and to determine the risk factors for the disease.
**Changhwan Kim M. D y col** [18]	2019	Cohort study	Corea	From 2013	Society	To estimate the direct and indirect costs and inform the establishment of efficient strategies to reduce the financial burden of COPD in Korea.
**Rehman A et al.** [19]	2019	Systematic review	USA, Asia and Europe	Until 2019	NHS	To find the economic burden of COPD in the U.S., Europe and Asia and identify key cost drivers in the treatment of COPD patients.
**Viinanen A et al.** [20]	2019	Cohort study	Finland	2004–2015	NHS	To describe healthcare resource utilization and associated costs in COPD patients in Finland according to disease severity and blood eosinophil count.
**Woo L et al.** [21]	2019	Systematic review	Asia and Pacific	2000–2018	Society	To evaluate COPD cost studies and analyze cost comparisons between Asia-Pacific countries.
**Iheanacho I et al.** [22]	2020	Systematic review	Worldwide	2006–2016	Society	To highlight the economic burden associated with moderate-severe COPD and to investigate the key drivers of healthcare resource utilization, direct and indirect costs.
**Rehman A et al.** [23]	2020	Systematic review	Europe	Until 2019	Society	To estimate the economic impact of COPD and identify key cost drivers in the treatment of COPD patients in different European countries.

*NHS: National Health System

As shown in Table 3 (methodological aspects of the articles), first, the variables used to estimate costs were identified. These were divided into several groups. Starting with those variables related to the sociodemographic characteristics of the patient and/or caregiver, mainly elements such as age, sex, smoking, employment status or socioeconomic status. Secondly, it is also important to highlight the presence, in most of the included studies, of variables that respond to patient characteristics related to the disease, mainly: GOLD stage, COPD severity, years of follow-up of the disease, respiratory symptoms or the CAT scale (COPD impact and quality of life test). This leads to the variables related to direct health care costs, mainly consisting of the costs of patients hospitalized for COPD and/or other causes, outpatient costs, drug costs or costs of visits to the emergency department. In relation to direct non-health costs, these mainly include the variables of the cost of home oxygen, medical transport, and informal care. Furthermore, there are variables that study indirect costs, in this case mainly related to loss of productivity and absenteeism, early retirement or premature mortality. Finally, we find the variables related to the study of intangible costs, where HRQOL and DALYs are identified.

**Table 3:** Methodological aspects of the articles: variables collected to estimate direct healthcare costs, direct non-medical costs, indirect costs and intangible costs, and limitations

Article	Variables collected	Limitations
**Foo J et al.** [6]	**Sociodemographic characteristics of the patient:** sex, age, years working, **Patient characteristics related to the disease:** COPD severity, mMrc, CAT and comorbidities. **Direct healthcare costs:** moderate and severe exacerbations, contact with health professionals, COPD medications, and flu vaccination in the past 12 months. **Indirect costs:** lost productivity and associated annual gain based on the above loss.	The different local unit costs of health services in each country had a limited impact on the variability between different countries. There is also wide variability among COPD patients surveyed in various countries. Direct costs are likely to depend on health systems and patient access to health care. Developed countries where access to care is often free tend to have higher costs. Other factors influencing variations in the data could be related to cultural differences and health practices.
**Wacker M. E et al.** [7]	**Sociodemographic characteristics of the patient:** age, sex, smoking and educational level. **Patient characteristics related to the disease:** FEV_1_, BMI, concomitant pathologies (MD, cerebrovascular accident (CVA), acute myocardial infarction (AMI), cancer and arthritis). **Direct healthcare costs:** visits to the doctor (last 3 months) and 13 specialties and prescribed drugs. **Indirect costs:** work absenteeism (last 12 months) and early retirement.	Regional restriction of the control group, possible selection bias that could have resulted in underestimation or overestimation of excess COPD costs, limiting the potential for external validation. Recall bias in interviews to record direct healthcare costs. Additional health services such as nursing or premature death costs were not considered. Cohort had to be free of moderate-severe exacerbations in the last 4 weeks before participation.
**Chen W et al.** [8]	**Sociodemographic characteristics of the patient:** age and sex. **Patient characteristics related to the disease:** comorbidities. **Direct healthcare costs:** inpatient costs, outpatient costs and prescribed drugs.	The impact of COPD severity on comorbidity costs was not assessed because information on pulmonary function measures in health data was missing. Lack of information from missing diagnosis registries. The study did not adjust cost estimates using survival for each year of follow-up.
**Tachkov K et al.** [9]	**Sociodemographic characteristics: of the patient** age and sex. **Patient characteristics related to the disease:** severity of COPD. **Indirect costs:** absenteeism (loss of productivity due to sick leave) and presenteeism (loss of productivity due to reduced work capacity), early retirement or permanent reduction in work capacity. **Intangible costs**: DALY.	Short follow-up period (1 year). Use of GDP per capita. In the calculation of DALY, YLL was assumed to be 0, because no patients died during the study period. This linked DALY directly to years of disability.
**Wacker M. E et al.** [10]	**Sociodemographic characteristics of the patient:** sex, age, smoking and educational level. **Patient characteristics related to the disease:** GOLD stage, spirometry variables, BMI, comorbidities, and symptoms (cough, expectoration, dyspnea, or no symptoms). **Direct healthcare costs:** outpatient costs, hospitalizations, and medication costs. **Indirect costs:** absence from work and early retirement.	The central data are based on self-reported information by study participants (recall bias). Consequently, the economic burden of COPD could be underestimated. History of exacerbations was not included as a possible cost factor. The design does not allow investigating whether COPD-related costs are increased by comorbid conditions.
**Quang Vo T et al.** [11]	**Sociodemographic characteristics of the patient:** age, sex and health insurance status. **Direct healthcare costs:** drugs and medical examinations, laboratory and functional tests and hospitalization costs.	The retrospective design may have led to loss of data. The data used were from a public hospital database; therefore, the results are primarily applicable to public care settings. The accuracy of diagnosis and management was sensitive to the diagnostic criteria used by the reporting physicians.
**Lakiang T et al.** [12]	**Sociodemographic characteristics of the patient:** age, marital status, employment status, number of family members, health insurance, monthly income, and educational level. **Direct healthcare costs:** visits by healthcare professionals, prescribed drugs, and hospitalizations. **Direct non-medical costs:** medical transportation and meals. **Indirect costs:** absolute absenteeism, relative absenteeism, work hours lost, absolute absenteeism and relative absenteeism.	The total sample size was not achieved, as many of the patients who were identified as subjects were retired or had not been working for more than one month, so they were excluded from the study. The sample size was compromised regarding the productivity and absenteeism study. The indirect cost was not reported in monetary terms.
**Lisspers K et al.** [13]	**Sociodemographic characteristics of the patient:** age and sex. **Patient characteristics related to the disease:** comorbidities and pharmacological treatment. **Direct healthcare costs:** COPD drugs, all drugs, nights hospitalized for COPD, nights hospitalized for other causes, hospital visits for COPD, hospital visits for other causes, doctor visits, visits by other health professionals. **Indirect costs:** loss of income in COPD patients.	The retrospective design of the study means that it may be subject to bias and confounding. The results for absolute cost have limited generalizability outside Sweden, as cost structures, prices, and supply differ between countries. Some sources of costs (personal care and others attributable to family caregivers) may have been overlooked The reference population was identified from patients without COPD who were required to attend the study’s Primary Care centers rather than healthy individuals from the general population.
**Merino M et al.** [14]	**Sociodemographic characteristics of the patient:** sex, age, smoking, educational level and employment category. **Patient characteristics related to the disease:** spirometry parameters, disease severity, exacerbations in the last 12 months, number and description of comorbidities and BMI. **Sociodemographic characteristics of the caregiver:** gender, age and relationship to the patient. **Direct healthcare costs:** medical tests (blood tests, pre-post spirometry, alpha-I anti-trypsin, X-rays, CAT scan and blood gas), pneumologist visits, Emergency Department visits, hospital admissions and prescribed drugs. **Direct non-medical costs:** permanent stay in a home or residential center, attendance at a day care center, professional home care, informal care, use of medical transportation, home oxygen, and number of informal caregivers. **Indirect costs:** labor productivity losses.	The severity level of COPD was missing in most (58.3%) of the participants’ medical records. Resource use corresponds to those associated with COPD in the last 12 months, but it is possible that a proportion is not strictly associated with COPD but with its related comorbidities. Recall bias in relation to work hours lost in the last 12 months of working patients (7.9%).
**Patel JG et al.** [15]	**Sociodemographic characteristics related to the patient:** age, sex, geographic region, type of plan, type of eligibility (leave + short-term disability, leave only or short-term disability only) and type of industry. **Patient characteristics related to the disease:** comorbidities. **Direct healthcare costs:** Assessments, hospitalizations, doctor’s visits, visits to other professionals and prescribed drugs. **Indirect costs:** absenteeism and short-term disability.	The small sample size limits the interpretation of the impact of exacerbation frequency on absenteeism in the current study. The models were adjusted for comorbidities included in the CCI, rather than a complete list of possible comorbidities, which means that some conditions may not have been considered. The indirect cost estimate only considers short-term disability and not absenteeism, presenteeism, or long-term disability. This may underestimate the costs to an employer. In addition, the short-term disability data were extracted from the MarketScan database whose purpose and consistency have not been confirmed. The exacerbator status assessment used medical claims to define exacerbator groups in the same period as the outcomes. This potentially confounded the impact of exacerbations on direct costs.
**Stafyla E et al.** [16]	**Sociodemographic characteristics of the patient:** age, sex, occupational exposure, employment status and smoking. **Patient characteristics related to the disease:** BMI, years with COPD, spirometry parameters, respiratory symptoms, comorbidities, GOLD stage and GOLD group. **Direct healthcare costs:** pharmacological treatment, medical visits, respiratory function tests, vaccinations, and laboratory tests. **Direct non-medical costs:** transport and home oxygen.	This study was designed to estimate the direct cost of COPD during the maintenance phase and not to assess the cost of exacerbations, which contributes a significant proportion of total cost in many studies. Due to the absence of related data, indirect costs could not be calculated. A large proportion of patients visited NHS units free of charge, so the cost of medical visits was underestimated. A final limitation could be the small population size.
**Zhu B et al.** [17]	The number of studies included in the review was 47 with an average quality assessment score of 7.70 out of 10. **Direct healthcare costs, direct non-medical care costs, indirect costs and intangible costs** were evaluated: 13 studies evaluated quality of life.	N/A*
**Changhwan Kim M. D et al.** [18]	**Direct healthcare costs:** Outpatient and inpatient care, emergency room visits and prescribed drugs. (Formal + informal) **Direct non-medical costs:** transportation, home care and nursing costs. **Indirect costs:** premature mortality and productivity losses.	Sample size. Patient survey data were used to estimate direct nonmedical and indirect costs for the first time in Korea. The survey data were entirely patient-reported (recall bias). An attempt was made to recruit patients from primary clinics to tertiary hospitals. Most of the sample were tertiary hospital patients, so direct medical costs per patient were higher in tertiary care hospitals than in primary and secondary care facilities. Overall costs may be overestimated.
**Rehman A et al.** [19]	The number of studies included in the review was 40. **Direct healthcare costs** were evaluated.	N/A
**Viinanen A et al.** [20]	**Sociodemographic characteristics of the patient:** age and sex. **Patient characteristics related to the disease:** BMI, spirometry parameters, comorbidities, years of disease follow-up and deaths due to COPD or other causes. **Direct healthcare costs:** medical visits (outpatient + emergency), hospitalization days, medical procedures and surgeries, and laboratory.	It is likely that costs associated with medical care were underestimated, since all hospital days were valued equally in the analyses, however, severe exacerbations of COPD are treated with costly treatment that could not be assessed in this study. Indirect costs were not considered. Data were taken from specialty care hospital records, patients with severe COPD were likely referred or their exacerbations treated in specialty care whereas patients with non-severe COPD would have had a reason to go to specialty care (diagnostic purposes), therefore patients with non-severe COPD in this study likely had higher associated health costs than non-severe patients treated at the Primary Care level. Risk of data collection from unconscious patients that may affect population size and other outcomes.
**Woo L et al.** [21]	The number of studies included in the review was 10. **Direct and indirect costs** were evaluated.	N/A
**Iheanacho I et al.** [22]	The number of studies included in the review was 73. **Direct healthcare costs** and **direct non-medical costs** (22 articles) and **indirect costs** (1 article) were evaluated.	N/A
**Rehman A et al.** [23]	The number of studies included in the review was 19. **Direct and indirect costs** were evaluated.	N/A

**Source.***Own elaboration*

*N/A ➔ Not applicable

*COPD: Chronic obstructive pulmonary disease

*mMrc: Modified scale of Medical Research Council

*CAT: COPD Assessment Test

*FEV1: Peak expiratory volume in the first second

*BMI: Body mass index

*MD: Mellitus diabetes

*CVA: Cerebrovascular accident

*AMI: Acute myocardial infarction

*DALY: Disability adjusted life years

*GDP: Gross domestic product

*GOLD: Global initiative of chronic obstructive lung disease

*NHS: National health service

This table shows the main limitations identified by the authors of the articles included in the monograph, with many of them agreeing on the difficulties identified. In summary, we can highlight the variability between countries (both in terms of patients and health services), cultural differences and differences in health practices, recall and selection bias (which can lead to an underestimation or overestimation of costs), difficulties in reaching the sample size, heterogeneity in designs between studies, differences in the level of COPD severity of the patients selected, and the non-inclusion of the costs of exacerbations in some of the articles. See Table 3.

As shown in Table 4, the direct healthcare costs (17 articles), are the most studied and are present or referred to in 17 out of 18 studies. These are followed by studies analyzing indirect costs (13 articles), direct non-health costs (8 articles) and finally, intangible costs (2 articles).

**Table 4:** Economic aspects of the articles: currency/year and estimated cost (direct healthcare costs, direct non-medical care costs, indirect costs, and intangible costs)

Article	Year of publication	Direct healthcare costs	Direct non-medical costs	Indirect costs	Intangible costs	Currency/year	Calculated cost
**Foo J et al.** [6]	2016	**YES**	**NO**	**YES**	**NO**	Dollars – N/A*	**Direct healthcare costs:** It ranged from US$ 504 (South Korea) to US$ 9981. **Indirect costs:** Ranged from $979 (RuYESa) to $20,844 (U.S.).
**Wacker M. E et al.** [7]	2016	**YES**	**NO**	**YES**	**NO**	Euros/2012	**Direct healthcare costs:** 2595 for COPD Grade 1, 3475 for Grade 2, 5955 for Grade 3 and 8924 for Grade 4. **Indirect costs:** 8.621 Grade 1, 9871 Grade 2, 16,550 Grade 3 and 27,658 Grade 4.
**Chen W et al.** [8]	2017	**YES**	**NO**	**NO**	**NO**	Canadian dollars /2015	**Direct medical costs:** per patient/year were CAD 5196 (€3668).
**Tachkov K et al.** [9]	2017	**NO**	**NO**	**YES**	**YES**	Euros – N/A	**Indirect costs:** average annual per patient €521.45. The average due to early retirement €25,000 (€6250 per patient) and for presenteeism €3750.64 per year. **Intangible costs:** a patient with mild COPD spends 0.62 years of his or her life in disability, moderate 6.00 and severe 9.00. This amounts to indirect costs of: €3.596,52; €34.204,01 and €51.332,20.
**Wacker M. E et al.** [10]	2017	**YES**	**NO**	**YES**	**NO**	Euros/2012	**Direct healthcare costs:** 7263 per patient (48.8% hospitalization, 34.1% prescribed medication, 11.7% medical and outpatient consultations and 7.4% rehabilitation and physiotherapy). **Indirect costs:** €23.298.
**Quang Vo T et al.** [11]	2018	**YES**	**NO**	**NO**	**NO**	Dollars /2017	**Direct healthcare costs:** ranged from $22.4 to $32.7 per outpatient visit and from $180.9 to $386.9 per inpatient visit.
**Lakiang T et al.** [12]	2018	**YES**	**YES**	**YES**	**NO**	Indian Rupee (Rs) – N/A	**Direct healthcare costs:** 29.885 ± 11.995,33 Rs. **Direct non-medical costs:** 7.441,25 ± 2.228,90 Rs. **Indirect costs:** The mean absenteeism observed in the last 28 days was 193.50 ± 33.62 h. The mean absolute absenteeism was 72.05 ± 7.55 h.
**Lisspers K et al.** [13]	2018	**YES**	**NO**	**YES**	**NO**	Euros/2013	**Direct costs:** €13.179. **Indirect costs: €**28,000 per patient.
**Merino M et al.** [14]	2018	**YES**	**YES**	**YES**	**NO**	Euros/2015	Average annual cost per patient of €3077. **Direct healthcare costs:** € 1.645 (43,8%). **Direct non-medical costs:** € 1.440 (38,3%). **Indirect costs:** € 672 (17,9%).
**Patel JG et al.** [15]	2018	**YES**	**NO**	**YES**	**NO**	USD/2010	**Direct healthcare costs:** $6246 patient/year. **Indirect costs**: Productivity loss was 5 more days/year of absence from work in COPD patients and an increase for short-term disability of $641. Direct costs for frequent and infrequent exacerbators were significantly higher than for nonexacerbators.
**Stafyla E et al.** [16]	2018	**YES**	**YES**	**NO**	**NO**	Euros – N/A	**Total direct cost:** The mean annual direct cost per stable disease was estimated at €1034.55 per patient, of which €222.94 corresponded to out-of-pocket payments. The annual cost ranged from €408.23 to €2041.89 according to GOLD stages (I-IV) and from €550.01 to €1480 according to GOLD groups (A-D).
**Zhu B et al.** [17]	2018	**YES**	**YES**	**YES**	**YES**	USD – N/A	**Total direct cost:** 499–1.930 USD per capita per year. **Direct medical costs:** 72–3.565 USD per capita per year. **Indirect costs:** 20–783 USD per capita per year **Intangible costs**: quality of life status is worse among patients with COPD than in patients without COPD and they are at a higher risk of suffering depression.
**Changhwan Kim M. D et al.** [18]	2019	**YES**	**YES**	**YES**	**NO**	USD – N/A	**Total costs:** 1245 million USD. **Direct healthcare costs:** 206.909 thousand USD (formal) AND 44.239 thousand USD (informal) **Direct non-medical costs:** USD 4262 thousand (transportation) and USD 486.3 million (nursing) **Indirect costs:** 407,771 thousand (lost productivity) and USD 96,153 thousand (premature deaths).
**Rehman A et al.** [19]	2019	**YES**	**NO**	**NO**	**NO**	USD – 2019	**Direct healthcare costs:** In the USA, the annual direct medical cost was $10,367 and the hospitalization cost was $6852. In Asia, the annual direct medical cost per patient in Iran, Korea and Singapore was $1544, $3077 and $2335, respectively. That of hospitalization in Iran, Korea, Singapore, India, China, and Turkey was $865, $1371, $1868, $296, $1477 and $1031, respectively. In Europe, the annual direct medical cost per patient was $11,787 (Norway), $10,552 (Denmark), $8644 (Germany), $8203 (Italy), $7760 (Sweden), $3190 (Greece), $1889 (Spain), $2162 (Belgium) and $2254 (Serbia).
**Viinanen A et al.** [20]	2019	**YES**	**NO**	**NO**	**NO**	Euros – N/A	**Direct healthcare costs:** 3300–3900 per patient per year. Severe disease €564 vs non-severe €319 (patient/year) Severe COPD patients with eosinophils €5215 vs non-eosinophilic COPD €4456 and €2538 in patients with unknown eosinophilic status (patient/year).
**Woo L et al.** [21]	2019	**YES**	**YES**	**YES**	**NO**	USD/2017	**Total costs:** Total annual social costs ranged from $4398 to $23,049 per capita in Japan and from $453 to $12,167 in South Korea. Remaining countries: $2700 (Singapore), $4000 (Taiwan), $3942 (China) and $1105 (Thailand).
**Iheanacho I et al.** [22]	2020	**YES**	**YES**	**YES**	**NO**	N/A	**Direct costs:** Most studies reported data from Europe or North America. Trends were observed in multiple studies due to higher direct costs being associated with increasingly severe COPD and/or a history of more frequent or severe exacerbations. **Indirect costs:** Not significant.
**Rehman A et al.** [23]	2020	**YES**	**YES**	**YES**	**NO**	Euros/2019	**Direct costs:** per patient per year was €10,701 (Norway), €9580 (Denmark), €7847 (Germany), €7448 (Italy), €7045 (Sweden), €2896 (Greece), €1963 (Belgium) and €2047 (Serbia). **Indirect costs:** Loss of productivity was highest in Germany €5735 and lowest in Greece €998. It was estimated at €4824 (Bulgaria), €2033 (Denmark) and €1298 (Sweden).

**Source.***Own elaboration*

*N/A ➔ Not applicable

The main structure presented by most of the studies starts with an analysis of direct health costs, followed by direct costs unrelated to health, and finally by indirect costs. As traditionally occurs in cost-of-illness studies, the least studied costs were the intangible costs, since only 2 of the 18 studies included these in their research. This is possibly due to the lack of information and records and the difficulty of their evaluation, quantification, and subsequent analysis, since it is very difficult to create a monetary value for aspects such as health-related quality of life (HRQoL) or disability.

In short, the heterogeneity of the studies makes it difficult to compare results. In this regard, the cost varies greatly depending on the countries studied, whether they have public or private health systems and whether they are conducted in developed or developing countries. In conclusion, a great variability was found in the costs reported in the studies, where the total cost per COPD patient varies strikingly between geographically distant areas.

Starting with direct health care costs, the data are analyzed and presented for the different geographical areas studied. Thus, in Europe (the geographic area where most of the studies in this monograph are set), this cost ranges from high-cost areas such as Norway at €10,701 (€/2019) per patient per year and Belgium at €1963 (€/2019) per patient per year. The average cost established among eight European countries (Norway, Denmark, Germany, Italy, Sweden, Greece, Belgium, and Serbia) for total direct costs per patient per year is €6182 (€/2019) [23]. Secondly, in the case of Asia, regarding the direct healthcare cost, no major significant differences were found among the developed Asian countries (Japan, South Korea, Singapore, Taiwan, and China). The average is estimated at approximately $9172 (USD/2017) [21]. Finally, in the USA, the figures remain between $9981 (USD - N/A) and $6246 (USD/2010) [15].

Considering the indirect costs (the second most studied), starting again with Europe, Rehman et al. [23], in their recent published systematic review of European studies established that the loss of productivity was highest in Germany with €5735 and the lowest in Greece with €998. This productivity loss was estimated at €4824 in Bulgaria, €2033 in Denmark and €1298 in Sweden. In the case of Asian countries, the total annual social costs ranged from $4398 to $23,049 per capita in Japan and from $453 to $12,167 in South Korea, $2700 in Singapore, $4000 in Taiwan, $3942 in China, and $1105 in Thailand [21]. Finally, Patel et al. [15] (2018) published in the USA that the loss of productivity was 5 more days/year of absence from work in patients with COPD and an increase in short-term disability of $641 compared to patients without the disease.

The following are direct non-health care costs, these types of costs are present in eight of the 18 articles analyzed in this review, many of which have already been included in the section on direct healthcare costs. There are some exceptions that report total direct costs, making no distinction between health and non-medical costs.

Finally, only two authors included intangible costs in their articles. In summary they established that patients with mild COPD spend 0.62 years of their life in disability, for those with moderate COPD this can represent as much as 6 years, and in the case of severe stages of the disease, this figure amounts to 9 years y reporting that the quality of life was worse among patients with COPD than in patients without COPD and also that they had a higher risk of depression during the course of the disease than patients who did not suffer from the disease. See Table 4.

## Discussion and conclusions

COPD is one of the main chronic diseases identified in developed countries. The latest epidemiological data indicate a high growth rate, which is projected even more intensely into the future, mainly related to population aging and smoking, which is present in western societies with worrying figures. Its identified morbimortality is one of the factors that disrupts the economic stability of health institutions, which is one of the basic pillars of a nation, and consequently increases the costs attributable to society at large [1, 2].

Regarding the characteristics of the selected articles (Table 2) and their methodological aspects (Table 3), it is important to note that the variability of costs in the reported results are largely a consequence of methodological divergences and research objectives that impact the type of costs in the way existing resources are identified, measured, valued and consumed by COPD patients in the various study settings. This variability may also be due in part to real differences between countries, such as epidemiological differences, differences in the sociodemographic characteristics of the patients included in the analyses, the main characteristics of the health systems in each area and even differences in the prevention and treatment of COPD and its complications, as well as the way professionals work and cultural differences.

In the case of the economic characteristics of the selected articles (Table 4), one of the limitations found was that health cost studies report their results in different currencies (apart from some intangible cost studies). As this systematic review comprises worldwide studies, each study was conducted in a different geographical setting, consequently presenting its results in different currencies (euros, Indian rupee, Canadian dollar, or US dollar), as well as computing this value in different years, from 2010 for the oldest study to 2019 for the most recent. This, in turn makes it difficult to equate and compare results unanimously, which generates discrepancies and heterogeneity when presenting and reflecting on the results found. Secondly, the studies identified in this review clearly show that there are not many studies that simultaneously estimate direct healthcare costs, direct non-medical care costs and indirect costs. Only three authors carried out such a study [12, 14, 18]. The scarce presence of non-medical costs, which refer to items such as the costs derived from personal care (formal and informal) and the transport or subsistence costs required by the disease, is noteworthy. Less than half of the studies identified incorporated this item in some way, and if they did, in most cases they complemented healthcare costs. However, none of them included the family cost related to the care of persons as a consequence of the disease. Also, it is important to mention that some studies are based on the perspective of COPD cost calculation and others show the total attributable costs in COPD patients (in this case older patients) since the costs generated by the comorbidities and peculiarities of each patient in the study sample are also included.

In view of the objectives of this monograph, it is important to mention that the 18 cost studies identified in this systematic review indicate that COPD significantly increases overall economic costs, thus generating significantly higher excess costs for people with COPD, compared to people without the disease. Regarding the factors that increase costs in COPD patients, after reading and analyzing the 18 articles included in the review, the main link that leads to the greatest increase in costs in this pathology is AECOPD (acute exacerbation of COPD), the main complication, which occurs more frequently and is greater with more severe COPD and cases that are worse controlled. Thus, the patients who generate the most costs within the disease are those who are in a moderate-severe stage of the disease and who, in addition, have the worst control of their pathology. Poor control of respiratory disease in general, and specifically in the COPD patient, is based on poor adherence to treatment and a lack of skill or technique in carrying out the prescribed pharmacological treatment. This, together with the presence of other risk factors such as smoking or obesity and factors external to the patient, such as environmental conditions or aging, lead to an increase in the number of exacerbations, which in most cases can lead to visits to the emergency department or even hospitalizations that generate high costs, which, in addition to the patient’s own comorbidities, can be lengthy. These comorbidities are the second most present factor which the studies refer to the most regarding the increase in costs, since patients with a greater number of chronic diseases generate increased costs, due to a greater consumption of drugs, greater hospital and outpatient visits, the performance of greater functional and laboratory tests, and, directly and indirectly affecting parameters that make up work losses, increased disability, loss of HRQOL or the need for the presence of an informal caregiver to support the patient’s basic care.

Finally, a breakdown by type of cost shows that all the authors reached the consensus that the main components of direct healthcare costs are hospitalizations (produced in most cases by AECOPD) and medication to treat these complications, whereas in general the other components (medical visits, emergency visits, diagnostic tests) are less relevant. Likewise, regarding indirect costs, the studies identified highlight the importance that work losses can have as a consequence of the disease. These are reported by more than half of the authors included in the literature review. This aspect entails not only a loss for the employer or society as a whole, but also a direct impact on the economic stability of the individuals facing the disease and, consequently, also on their families or persons on whom their economic income depends.

In conclusion, given the importance of COPD, both for the health system and for society, it would be advisable to reassess the allocation of resources for the disease by taking into account the study of costs.

## Data Availability

The corresponding author on behalf of the other authors guarantee the accuracy, transparency and honesty of the data and information contained in the study, that no relevant information has been omitted and that all discrepancies between authors have been adequately resolved and described.
